# Transcriptome analysis of the gene expression of M*.* iliotibialis lateralis affected by dietary methionine restriction

**DOI:** 10.3389/fphys.2023.1184651

**Published:** 2023-05-22

**Authors:** Desheng Li, Fei Chen, Yumin Tian, Yuhong Su

**Affiliations:** ^1^ College of Animal Science and Veterinary Medicine, Jinzhou Medical University, Jinzhou, China; ^2^ Laboratory of Quality and Safety of Animal Product of Liaoning Province, Jinzhou, China; ^3^ College of Food and Health, Jinzhou Medical University, Jinzhou, China

**Keywords:** methionine, broiler chicken, skeletal muscle, transcriptome analysis, RNA-Seq

## Abstract

**Introduction:** Methionine (Met) is an important amino acid related to the development of skeletal muscle. This study investigated the effects of dietary Met restriction on the gene expression of M. iliotibialis lateralis.

**Methods:** A total of 84 day-old broiler chicks (Zhuanghe Dagu) with a similar initial body weight (207.62 ± 8.54 g) were used in this study. All birds were divided into two groups (CON; L-Met) based on the initial body weight. Each group consisted of six replicates with seven birds per replicate. The experimental period was 63 days (phase 1, days 1-21; phase 2, days 22-63). According to the nutritional requirements of Zhuanghe Dagu chickens, we provided a basal diet (0.39% Met levels during phase 1 and 0.35% Met levels during phase 2, as-fed basis) to the birds in the CON group, while we provided a Met-restricted diet (0.31% Met levels during phase 1 and 0.28% Met levels during phase 2, as-fed basis) to the birds in the L-Met group. The growth performance of broiler chicks and their M. iliotibialis lateralis development parameters were measured on days 21 and 63.

**Results and Discussion:** In this study, dietary Met restriction did not affect the growth performance of broiler chicks but hindered the development of M. iliotibialis lateralis at both sampling timepoints. On the final day, three birds selected from each group (three from CON and three from L-Met) were used to obtain M. iliotibialis lateralis samples from leg muscle for further transcriptome analysis. Transcriptome analysis revealed that dietary Met restriction significantly upregulated 247 differentially expressed genes (DEGs) and downregulated 173 DEGs. Additionally, DEGs were mainly enriched in 10 pathways. Among DEGs, we observed that dietary Met restriction downregulated the expression of *CSRP3*, *KY*, *FHL1*, *LMCD1*, and *MYOZ2* in M. iliotibialis lateralis. Therefore, we considered that dietary Met restriction had negative effects on the development of M. iliotibialis lateralis, and *CSRP3*, *KY*, *FHL1*, *LMCD1*, and *MYOZ2* may serve as potential functional genes involved in this process.

## Introduction

Methionine (Met), the first limiting amino acid for poultry, is closely related to the development of skeletal muscle in broiler chicks (Wen et al., 2014). Skeletal muscle is an important endocrine organ, secreting a series of bioactive substances with developmental and metabolic regulation characteristics via autocrine and paracrine pathways (Pedersen et al., 2004). Well development of skeletal muscle for poultry is not only beneficial to their health but also related to animal welfare (Dang et al., 2022a; Dang et al., 2022b). It is fully documented that dietary Met restriction hindered the development of skeletal muscle (Corzo et al., 2006; Wen et al., 2014).

M. iliotibialis lateralis, the largest muscle in the pelvic limb, plays an important role in supporting the animal and maintaining posture (Roy et al., 2007). M. iliotibialis lateralis is therefore selected to be used in this study to investigate the effects of dietary Met restriction on its development situation. Additionally, to understand the basic molecular mechanisms involved in this process, we conducted a transcriptome analysis for M. iliotibialis lateralis. Transcriptome sequencing technology could accurately and efficiently obtain almost all the transcripts of a specific tissue in a certain period of time and deeply excavate the subtle changes in the differential expression of genes in the tissue (Haas and Zody, 2010). Therefore, the transcriptome sequencing technique is of great significance for exploring gene expression and regulation mechanisms at the transcription level (Luo et al., 2021; Zhang et al., 2023).

Currently, the molecular mechanism of dietary Met restriction on skeletal muscle development is still unknown. We hypothesized that the gene expression profile of M. iliotibialis lateralis will change with dietary Met restriction, and therefore hindered its development. Therefore, the objective of this study was to investigate the effects of dietary Met restriction on growth performance, M. iliotibialis lateralis development parameters, and M. iliotibialis lateralis gene expression profile.

## Materials and methods

### Experimental design

A total of 84 day-old broiler chicks (Zhuanghe Dagu) with a similar initial body weight (207.62 ± 8.54 g) were used in this study. All birds were divided into two groups (CON; L-Met) based on their initial body weight. Each group consisted of six replicates with seven birds per replicate. The experimental period was 63 days (phase 1, days 1–21; phase 2, days 22–63). Based on the nutritional requirements of Zhuanghe Dagu chicken recommended by Tian (2014), we provided two kinds of diet to experimental birds (Table 1): birds in the CON group were fed with a basal diet (0.39% Met levels during phase 1 and 0.35% Met levels during phase 2, as-fed basis), while birds in the L-Met group were fed with a Met-restricted diet (0.31% Met levels during phase 1 and 0.28% Met levels during phase 2, as-fed basis).

**TABLE 1:** Composition and nutrient levels of the experimental basal diet (%, as-fed basis).

	Phase 1 (days 1–21)		Phase 2 (days 22–63)	
Ingredients, %	CON	L-Met	CON	L-Met
Corn	62.06	62.06	68.50	68.50
Soybean meal	30.77	30.77	26.11	26.11
Dicalcium phosphate	1.71	1.71	1.42	1.42
Limestone	1.65	1.65	1.29	1.29
NaCl	0.25	0.25	0.25	0.25
Sodium bicarbonate	0.19	0.19	0.19	0.19
Soy oil	2.19	2.19	1.09	1.09
Vitamin and trace mineral premix^a^	1.00	1.00	1.00	1.00
L-Cystine	0.08	0.16	0.07	0.14
DL-Methionine	0.10	0.02	0.07	-
L-Lysine HCl	-	-	0.01	0.01
Analyzed value, %				
Metabolizable energy, MJ/kg	12.31	12.26	12.30	12.29
Crude protein	19.01	18.97	17.48	17.39
Calcium	1.05	1.04	0.86	0.84
Phosphorus	0.40	0.39	0.36	0.35
Lysine	0.96	0.95	0.86	0.85
Methionine	0.39	0.31	0.35	0.28
Methionine + cysteine	0.79	0.77	0.74	0.73

^a^ Provided per kg of complete diet: 80 mg Fe; 8 mg Cu; 100 mg Mn; 80 mg Zn; 0.7 mg I; 0.3 mg Se; 6,500 IU of vitamin A; 850 IU of vitamin D_3_, 12.5 IU of vitamin E, 0.85 mg of vitamin K_3_, 0.7 mg of vitamin B_1_; 4.5 mg of vitamin B_2_; 9.2 mg of vitamin B_5_; 28 mg of niacin; 2.8 mg of vitamin B_6_; 0.14 mg of biotin; 0.6 mg of folic acid; 0.012 mg of vitamin B_12_; and 950 mg of choline.

Broiler chicks were caged in two-tier battery cages under a 16 h light/8 h dark cycle. The size of the steel cage was 70.5 × 40 × 42 cm (940 cm^2^ bird^−1^). Water and feed were provided *ad libitum* during the experimental period. The protocol of this study was proposed in accordance with the ARRIVE guidelines (https://arriveguidelines.org) for the reporting of animal experiments and was reviewed and approved by the Jinzhou Medical University Animal Care and Use Committee. All methods were performed in accordance with the relevant guidelines and regulations (201720528).

### Sampling and measurements

#### Body weight analysis

Cage-based body weight was recorded on days 1, 21, and 63 to calculate body weight gain (BWG). Cage-based feed intake was recorded weekly to calculate feed intake (FI). The feed-to-gain ratio was calculated using the value of BWG and FI.

#### M. iliotibialis lateralis parameter analysis

On days 21 and 63, two birds per replicate were randomly selected according to the mean body weight of broiler chick flock to obtain M. iliotibialis lateralis. On the sampling day, birds were euthanized with 1 cc Euthasol^®^ intravenously. M. iliotibialis lateralis located pre- and post-acetabularis (Rose et al., 2016) was removed and weighed to calculate the relative weight.

#### Transcriptome analysis of M. iliotibialis lateralis

On the final day, after measuring the weight of M. iliotibialis lateralis, three M. iliotibialis lateralis samples obtained from each group were immediately stored at −196°C in liquid nitrogen for further transcriptome analysis.

Approximately 0.2 g of M. iliotibialis lateralis tissue was used to extract total RNA using TRIzol reagent. The degree of RNA degradation was analyzed by agarose gel electrophoresis, and RNA purity was detected using a NanoDrop 2000 spectrophotometer. The RNA concentration was accurately quantified using Qubit 2.0, and RNA integrity was detected using the Agilent 2100 Bioanalyzer. Following sample testing, a measure of 3 µg RNA per sample was used as an input material for the RNA sample preparations. Sequencing libraries were generated using NEBNext^®^ Ultra™ RNA Library Prep Kit for Illumina^®^ (NEB, United States) following the manufacturer’s recommendations, and index codes were added to attribute sequences to each sample (Wang and Ma, 2019). The quality of library was assessed on the Agilent Bioanalyzer 2100 system.

The library preparations were sequenced on an Illumina HiSeq 2500 platform. The quality control of the reads was performed using in-house written scripts. Raw data of the FASTQ format were initially processed by in-house Perl scripts. In this step, clean reads were obtained by removing reads containing adapter, poly-N, and low-quality reads from raw data. Q20, Q30, and GC content were calculated for the clean data. All downstream analyses were based on clean data with high quality. The PE 150 paired-end sequencing strategy was used in this study. The chick’s genome sequence (90 version) was downloaded from the genome website (ftp://ftp.ensembl.org/pub/current_fasta/gallus_gallus/dna/Gallus_gallus.Gallus_gallus-5.0.dna.toplevel.fa.gz). The index of the reference genome was built using HISAT2 v2.0.5, and paired-end clean reads were aligned to the reference genome using HISAT2 v2.0.5. The gene expression level was estimated by using the number of normalized fragments per kilogram of transcript per million fragments (FPKM) method. The differential expression analysis of the groups was performed using the DESeq 2R package (1.16.1) based on the readcount data. Pathway enrichment analysis was assessed using the Kyoto Encyclopedia of Genes and Genomes (KEGG) database (http://www.genome.jp/kegg/). The clusterProfiler R package was used to test the statistical enrichment of differential expression genes in KEGG pathways.

#### qRT-PCR verification

The expression of *CSRP3*, *KY*, *FHL1*, *LMCD1*, and *MYOZ2* genes was measured by qRT-PCR to verify the accuracy of transcriptome sequencing data by RNA-Seq. After extracting total RNA from hepatic tissue samples, cDNA was synthetized using the total RNA reverse transcriptase kit (Takara, Dalian, China). Real-time PCR was performed on an ABI 7500 Fast Real-Time PCR system using SYBR premix Ex Taq™ Ⅱ (Takara). The optimized cycling conditions were as follows: denaturation at 94°C for 5 min followed by 45 cycles of 94°C for 15 s and 55°C for 15 s. Each sample was tested in triplicate. The relative expression was determined using the 2^−ΔΔCt^ method (Schmittgen and Livak, 2008), and β-actin was used as the internal control for normalization of the results. The sequences of primers for the genes tested were specifically designed according to the sequences located in GenBank (Table 2).

**TABLE 2:** Primers used for quantitative real-time PCR.

Gene	Primer sequence (5’→3′)	
*β-Actin*	Forward	GCCCAGCACGATGAAGAT
	Reverse	ATT​TAC​GGT​GGA​CGA​TGG​AC
*CSRP3*	Forward	CAGTTCATTTCGTTCCC
	Reverse	AGGTCATTCAGGTGGTC
*KY*	Forward	GGTTTGGATAGGGTAAG
	Reverse	GTGTTCTGTCGTCTGGA
*FHL1*	Forward	CAATCGTCGTCAGGA
	Reverse	GAGGAAAAGACAGTGC
*LMCD1*	Forward	GTCTGACTATGCGGAGTT
	Reverse	CGTAAGGGCGGAAGG
*MYOZ2*	Forward	TAAGATGCGTCAAAGA
	Reverse	AAGTTCCTCAGTGCC

*CSRP3*, cysteine- and glycine-rich protein 3; *KY*, kyphoscoliosis peptidase; *FHL1*, four and a half LIM domains 1; *LMCD1*, LIM- and cysteine-rich domains 1; *MYOZ2*, myozenin 2.

### Statistical analysis

The normality of growth performance and M. iliotibialis lateralis parameter were examined by the Shapiro–Wilk test and quantile–quantile plot, respectively. Then, data were analyzed by the one-way ANOVA model with Dunnett’s *post hoc* test using SPSS software (version 26.0). The results were presented as the means ± standard deviation. The probability value below 0.05 was considered statistically significant.

## Results and discussion

Met, the first limiting amino acid for poultry, is closely related to their growth (Bunchasak, 2009). Providing diets that do not meet the Met requirement for poultry will hinder their growth performance (Fagundes et al., 2020). Fagundes et al. (2020) assigned 40 broiler chicks into Met-sufficient or Met-deficient groups and found that birds fed with Met-deficient diet had worse growth and feed efficiency. Liu et al. (2022) also provided chicks with Met-deficient diet and reported that body weight, weight gain, and feed efficiency were impaired by restricting dietary Met contents. However, in this study, we did not observe that dietary Met restriction had negative effects on BWG, FI, and feed efficiency of broiler chicks (Table 3). The differences in the results compared to the aforementioned studies may be due to the use of native breed of experimental animal. In comparison to the commercial breed of chicks, the native breed of chick has different nutritional requirements.

**TABLE 3:** Growth performance of broiler chicks as affected by dietary methionine restriction.

Item	CON^a^	L-Met^b^
BWG, g		
Days 1–21	445.24 ± 4.76	443.81 ± 41.80
Days 22–63	1385.44 ± 120.40	1315.00 ± 137.62
Days 1–63	1830.68 ± 122.90	1758.81 ± 179.06
FI, g		
Days 1–21	1443.33 ± 80.22	1421.91 ± 78.96
Days 22–63	4150.44 ± 231.00	4380.60 ± 243.18
Days 1–63	5277.51 ± 293.58	5419.26 ± 300.51
Feed-to-gain ratio		
Days 1–21	0.31 ± 0.01	0.31 ± 0.03
Days 22–63	0.33 ± 0.03	0.30 ± 0.03
Days 1–63	0.35 ± 0.02	0.33 ± 0.03
The data are presented as the means ± standard deviation		
BWG, body weight gain; FI, feed intake		

^a^ Birds in the CON group were fed a basal diet (0.39% Met levels during days 1–21 and 0.35% Met levels during days 22–63, as-fed basis).

^b^ Birds in the L-Met group were fed a Met-restricted diet (0.31% Met levels during days 1–21 and 0.28% Met levels during days 22–63, as-fed basis).

However, we observed a decrease in the relative weight of M. iliotibialis lateralis induced by dietary Met restriction on days 21 (*p* < 0.05) and 63 (*p* < 0.05) (Table 4). Met is the initial amino acid in protein synthesis for eukaryotes. Met restriction will reduce muscle anabolism and increase catabolism, thus impairing protein synthesis in muscle (Zeitz et al., 2019; Ghavi et al., 2020). It is reported that dietary Met restriction aggravated the muscle atrophy induced by denervation (Swaminathan et al., 2021) and increased the rate of muscle protein degradation (Barnes et al., 1995). On the other hand, dietary Met restriction resulted in insulin resistance (Ying et al., 2019). Insulin resistance could be considered a contributor to muscle wasting (Guillet and Boirie, 2005). Therefore, the results obtained in this study proved that dietary Met restriction had negative effects on M. iliotibialis lateralis development.

**TABLE 4:** Relative weight of M. iliotibialis lateralis of broiler chicks as affected by dietary methionine restriction.

Item, %	CON^a^	L-Met^b^
Day 21	0.93 ± 0.06^a^	0.84 ± 0.09^b^
Day 63	0.94 ± 0.03^a^	0.85 ± 0.08^b^

The data are presented as the means ± standard deviation.

^a,b^Different superscripts within a row indicate a significant difference (*p* < 0.05).

^a^ Birds in the CON group were fed a basal diet (0.39% Met levels during days 1–21 and 0.35% Met levels during days 22–63, as-fed basis).

^b^ Birds in the L-Met group were fed a Met-restricted diet (0.31% Met levels during days 1–21 and 0.28% Met levels during days 22–63, as-fed basis).

**FIGURE 1:**
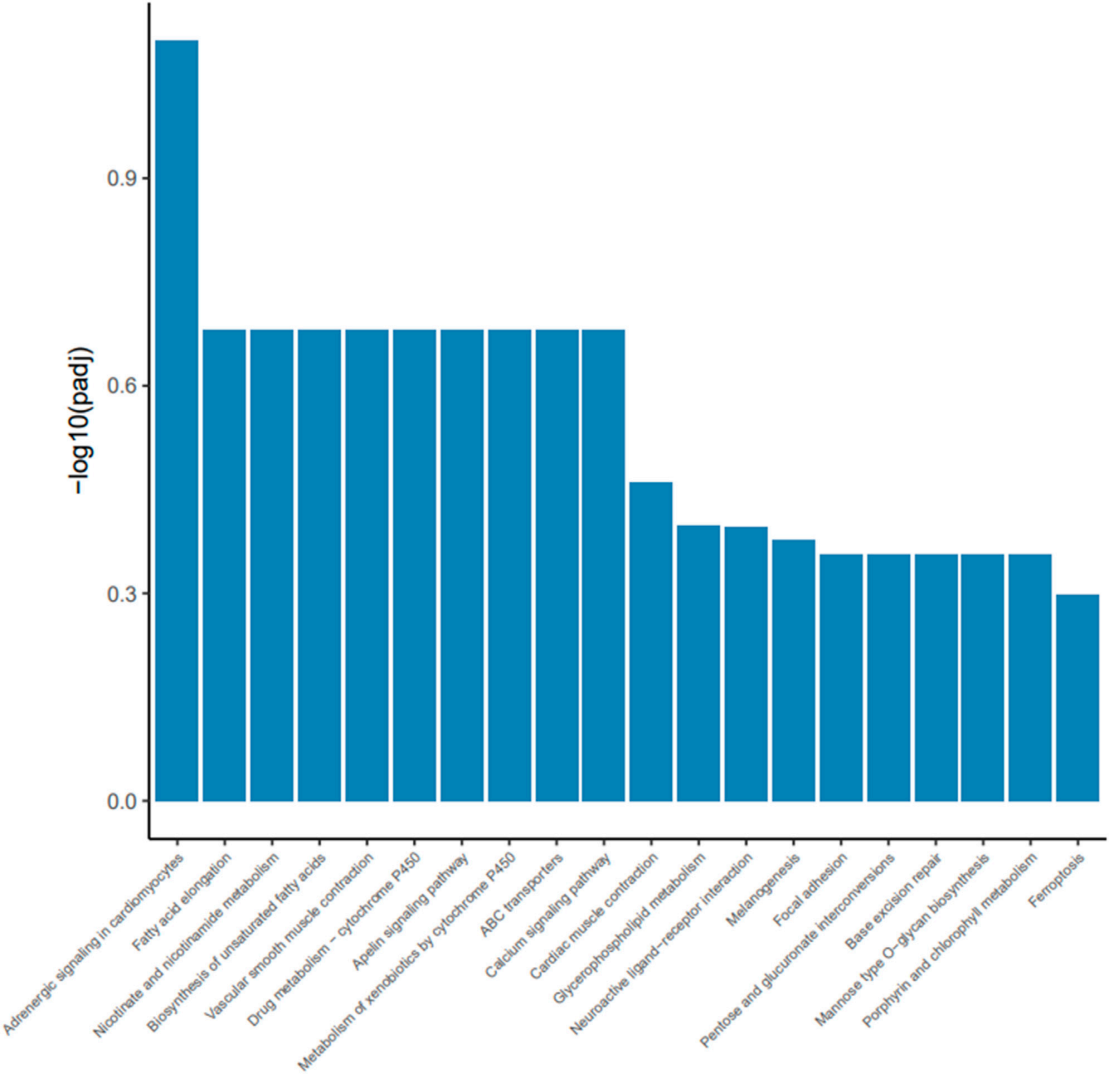
Advanced bubble chart shows significantly enriched pathways based on differentially expressed genes (DEGs) by Kyoto Encyclopedia of Genes and Genomes pathway analysis (*p* < 0.05). The *x*-axis represents rich factor (rich factor = number of DEGs enriched in the pathway/number of all genes in the background gene set). The *y*-axis represents the enriched pathway. Color represents enrichment significance, and the size of the bubble represents the number of DEGs enriched in the pathway.

To investigate how dietary Met restriction affects the gene expression of M. iliotibialis lateralis, we further conducted a transcriptome analysis for M. iliotibialis lateralis*.* A total of 11.29 Gb and 10.82 Gb clean reads were obtained from different groups, respectively, and the value of Q20 in both groups was higher than 96% and that of Q30 was higher than 90%. Additionally, the GC contents in both groups were higher than 55% (Table 5). As expected, we observed that 247 DEGs (75 known genes) were downregulated, while 173 DEGs (63 known genes) were upregulated, by dietary Met restriction (Supplementary file). Pathway enrichment analysis showed that DEGs in M. iliotibialis lateralis mainly enriched in adrenergic signaling in cardiomyocytes, fatty acid elongation, nicotinate and nicotinamide metabolism, biosynthesis of unsaturated fatty acids, vascular smooth muscle contraction, drug metabolism—cytochrome P450, apelin signaling pathway, metabolism of xenobiotics by cytochrome P450, ABC transporters, and calcium signaling pathway (*p* < 0.05) (Figure 1). Among them, adrenergic signaling, nicotinate and nicotinamide metabolism, biosynthesis of unsaturated fatty acids, and calcium signaling pathway are important pathways associated with the skeletal muscle development. Adrenergic signaling is associated with the anabolic effects in skeletal muscle, and the stimulation of the β-adrenoceptors in skeletal muscle is considered an effective measure to therapy skeletal muscle wasting disorders (Lynch and Ryall, 2008). Oxidized nicotinamide adenine dinucleotide plays a beneficial role in promoting muscle development and maintaining muscle health (Goody and Henry, 2018). Unsaturated fatty acids can promote muscle fiber development (Wang et al., 2022). Ca^2+^ is an important component of the signaling, promoting muscle formation, muscle homeostasis, and regeneration (Tu et al., 2016). Moreover, the elongation of fatty acid in muscle (Wang et al., 2022) and apelin signaling (Frier et al., 2009) are closely related to the morphology and biogenesis of mitochondria in muscle. The smooth muscle cell (Brozovich et al., 2016) and cytochrome P450 (Hillig et al., 2003) are important to regulate the size of the blood vessel lumen and blood flow. ABC transporters facilitate the transport of various endogenous substances, as well as substances foreign to the body (Annaert et al., 2001). Therefore, pathway enrichment analysis indicated that dietary Met restriction had regulating effects on the development of M. iliotibialis lateralis.

**TABLE 5:** Statistics of sequencing data.

Item	Clean reads	Clean base (G)	Q20, %	Q30, %	GC content, %
CON^a^	3,764,3189	11.29	96.49	91.24	56.15
L-Met^b^	36,061,932	10.82	96.78	92.00	56.03

^a^ Birds in the CON group were fed a basal diet (0.39% Met levels during days 1–21 and 0.35% Met levels during days 22–63, as-fed basis).

^b^ Birds in the L-Met group were fed a Met-restricted diet (0.31% Met levels during days 1–21 and 0.28% Met levels during days 22–63, as-fed basis).

**FIGURE 2:**
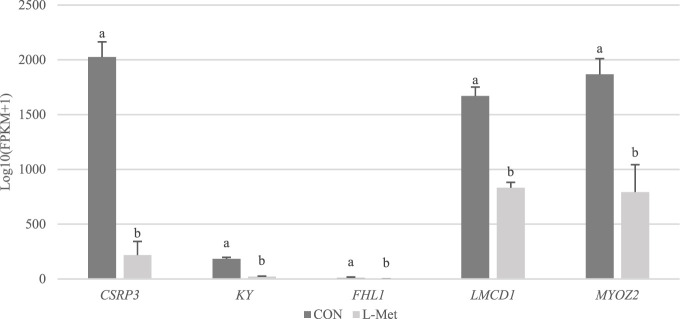
Comparison of transcripts per million values of *CSRP3*, *KY*, *FHL1*, *LMCD1*, and *MYOZ2* genes between CON and L-Met groups.

**FIGURE 3:**
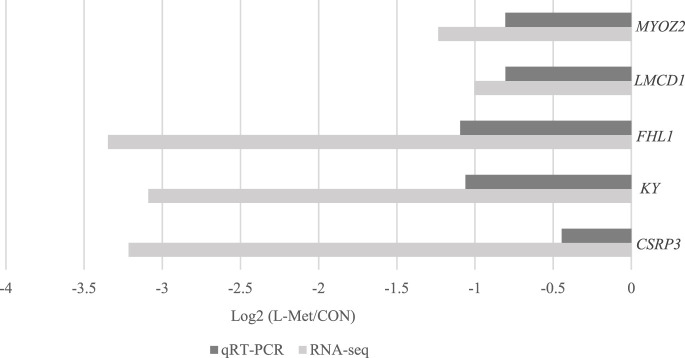
Comparison of *CSRP3*, *KY*, *FHL1*, *LMCD1*, and *MYOZ2* expression between qRT-PCR and RNA-Seq.

Among DEGs, only *CSRP3*, *KY*, *FHL1*, *LMCD1*, and *MYOZ2* were known genes related to skeletal muscle development, which were downregulated by dietary Met restriction (*p* < 0.05) (Figure 2). To verify the results of RNA-Seq, qRT-PCR was used to perform *CSRP3*, *KY*, *FHL1*, *LMCD1*, and *MYOZ2* expression. The expression profiles of these genes corresponded to the RNA-Seq results (Figure 3). The protein encoded by *CSRP3* is a positive regulator for myogenesis (Cui et al., 2020). The length of sarcomere and muscle fiber of skeletal muscle in *CSRP3* gene-deficient mice is shorter than that in normal mice (Barash et al., 2005). Interfering with the expression of *CSRP3* will inhibit the differentiation of chicken skeletal muscle satellite cells into myotubes and therefore hinder the development of skeletal muscle (Han et al., 2019). *KY* gene encodes the protein belonging to the transglutaminase-like superfamily, which is involved in the function, maturation, and stabilization of the neuromuscular junction, and is required for normal muscle growth (Vargas et al., 2002). Muscle hypertrophy in the *KY* gene mutant mouse is defective (Blanco et al., 2001). *FHL1* is suggested to play a role in sarcomere synthesis and assembly (McGrath et al., 2006). *FHL1* has been shown to be involved in regulating muscle fiber type I development (Chauvigné et al., 2005). *LMCD1* gene plays a critical role in the development of muscle hypertrophy via activation of the calcineurin/nuclear factor of the activated T-cell signaling pathway (Bian et al., 2010). Moreover, *MYOZ2* plays a role in myofibrillogenesis (Takada et al., 2001). Therefore, we speculated that *CSRP3*, *KY*, *FHL1*, *LMCD1*, and *MYOZ2* may serve as functional genes involved in M. iliotibialis lateralis development whose hindering was induced by dietary Met restriction.

In conclusion, this study demonstrated that dietary Met restriction had negative effects on the development of M. iliotibialis lateralis, which may be achieved by regulating the pathways of adrenergic signaling, nicotinate and nicotinamide metabolism, biosynthesis of unsaturated fatty acids, and calcium signaling pathway. Additionally, *CSRP3*, *KY*, *FHL1*, *LMCD1*, and *MYOZ2* may serve as functional genes involved in this process.

## Data Availability

The datasets generated and/or analyzed during the current study are available in the Figshare repository, https://doi.org/10.6084/m9.figshare.22045055.v1.

## Abbreviations

Met, methionine; ADG, average daily gain; ADFI, average daily feed intake.
